# Pneumatosis intestinalis and porto-mesenteric venous gas: a multicenter study

**DOI:** 10.1186/s12880-021-00651-y

**Published:** 2021-08-24

**Authors:** Marta Della Seta, Roman Kloeckner, Daniel Pinto dos Santos, Thula Cannon Walter-Rittel, Felix Hahn, Jörn Henze, Annika Gropp, Johann Pratschke, Bernd Hamm, Dominik Geisel, Timo Alexander Auer

**Affiliations:** 1 Department for Radiology, Charité - University Medicine Berlin, Campus Virchow-Klinikum, Augustenburger Platz 1, 13353 Berlin, Germany; 2 Department for Radiology, University Hospital Mainz, Mainz, Germany; 3 Department for Radiology, University Hospital of Cologne, Cologne, Germany; 4 Department of General, Visceral and Transplant Surgery, Charité – University Medicine Berlin, Berlin, Germany; 5 Berlin Institute of Health (BIH), Berlin, Germany

**Keywords:** Pneumatosis intestinalis, Porto-mesenteric venous gas, PMVG, Bowel ischemia, Sepsis

## Abstract

**Background:**

Estimating the prognosis of patients with pneumatosis intestinalis (PI) and porto-mesenteric venous gas (PMVG) can be challenging. The purpose of this study was to refine prognostication to improve decision making in daily clinical routine.

**Methods:**

A total of 290 patients with confirmed PI were included in the final analysis. The presence of PMVG and mortality (90d follow-up) were evaluated with regard to the influence of possible risk factors. Furthermore, a linear estimation model was devised combining significant parameters to calculate accuracies for predicting death in patients undergoing surgery by means of a defined operation point (ROC-analysis).

**Results:**

Overall, 90d mortality was 55.2% (160/290). In patients with PI only, mortality was 46.5% (78/168) and increased significantly to 67.2% (82/122) in combination with PMVG (median survival: PI: 58d vs. PI and PMVG: 41d; *p* < 0.001). In the entire patient group, 53.5% (155/290) were treated surgically with a 90d mortality of 58.8% (91/155) in this latter group, while 90d mortality was 51.1% (69/135) in patients treated conservatively. In the patients who survived > 90d treated conservatively (24.9% of the entire collective; 72/290) PMVG/PI was defined as “benign”/reversible. PMVG, COPD, sepsis and a low platelet count were found to correlate with a worse prognosis helping to identify patients who might not profit from surgery, in this context our calculation model reaches accuracies of 97% specificity, 20% sensitivity, 90% PPV and 45% NPV.

**Conclusion:**

Although PI is associated with high morbidity and mortality, „benign causes” are common. However, in concomitant PMVG, mortality rates increase significantly. Our mathematical model could serve as a decision support tool to identify patients who are least likely to benefit from surgery, and to potentially reduce overtreatment in this subset of patients.

## Key points

**Key Point 1**: The coincidence of porto-mesenteric venous gas (PMVG) and pneumatosis intestinalis (PI) is indicative for a significant increase of mortality.

**Key Point 2**: PMVG, COPD, sepsis and a low platelet count were found to correlate with a worse prognosis helping to identify patients who might not profit from surgery.

**Key Point 3**: Our linear regression model reached a specificity of 97% in predicting death for patients undergoing surgery and might thus be helpful in clinical decision-making.

## Introduction

Pneumatosis intestinalis (PI) is defined as the presence of gas within the submucosal or mucosal wall of the intestine [1]. PI was first described anatomically in 1730 by *Duvernoy* and named nearly hundred years later by *Mayer* as pneumatosis cystoides intestinalis [1–3]*.* PI is a radiographic or histopathologically verified phenomenon, rather than a clinical diagnosis with potentially life-threatening consequences for the patient. The aetiology of PI is complex. But overall, three causes have been identified as the source/origin of gas within the intestinal wall:

1. gas formed in the intestine, i.e. luminal gas [4].
2. gas-producing bacteria [5, 6].
3. gas from the respiratory system [7–9].

Aetiology may range from ischemia or bowel obstruction to sepsis and many other conditions. Apart from these clearly life-threatening pathologies, benign/reversible causes like respiratory disorders and / or unfavourable drug related side effects—e.g. chemotherapy-associated—have been reported [10–12]. The Clinical presentation can be unspecific, but the most common symptoms involve abdominal pain, diarrhoea, bloody stools, fever, and shock [1, 13, 14]. Upon presentation, a clinical examination must be performed, and abdominal x-ray can be helpful. However, in clinical reality, computed tomography (CT) is the modality of choice for diagnosing PI [3, 15].

**Fig. 1:**
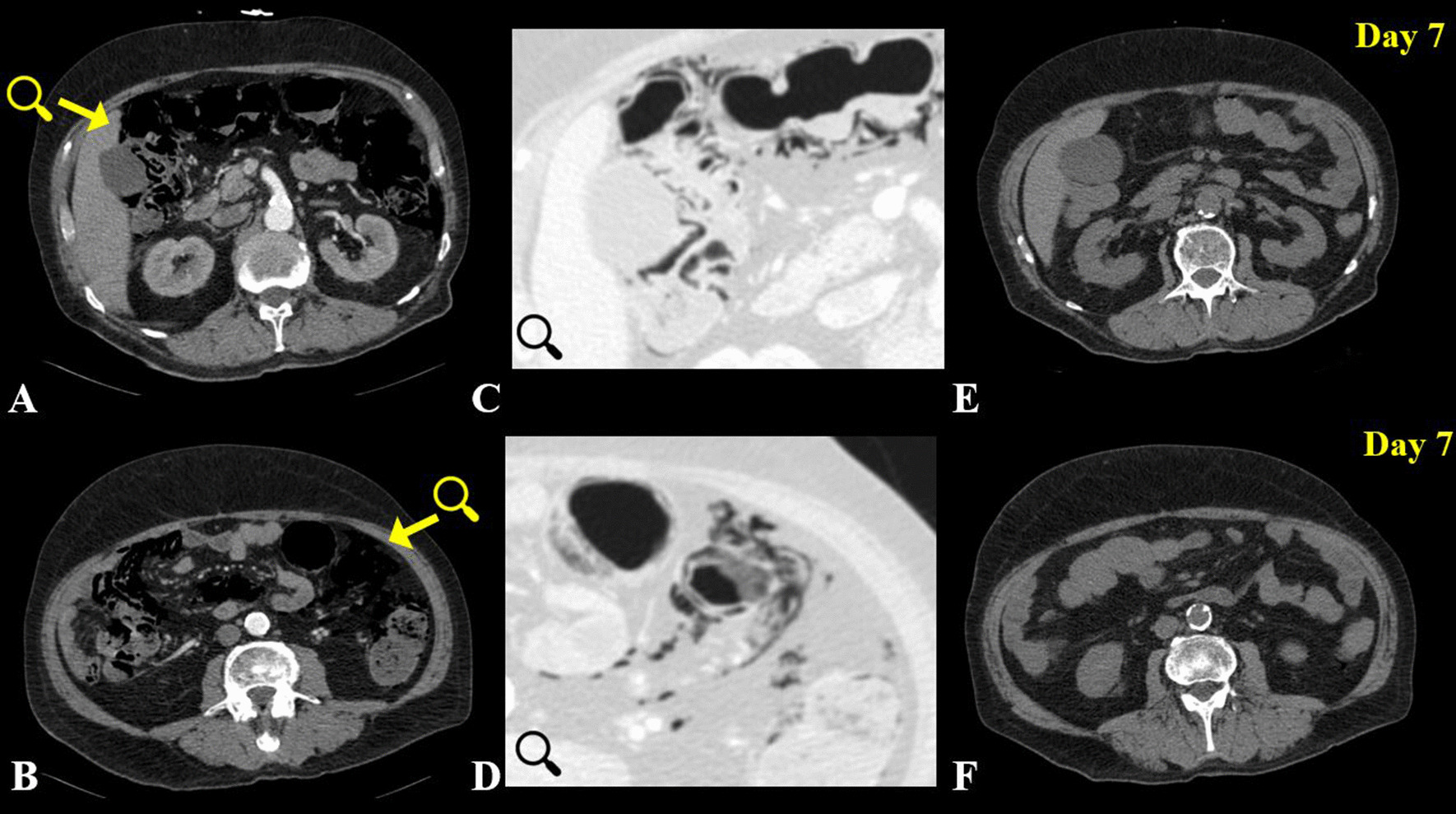
Arterial phase Contrast enhanced axial CT with remarkable PI and pronounced mesenteric gas (**A**, **B**) in a 73-year-old patient without intestinal necrosis or ischemia. **C**, **D** are illustrating (magnified) the intestinal segments the yellow arrows in **A** and **B** are pointing at but in a lung window to emphasize the extent and localization of the gas. PI was fully reversible upon seven-day follow-up non contrast enhanced axial CT (**E**, **F**). As the patient was receiving chemotherapy for cancer of the hypopharynx, this may be a case of drug-related reversible/ “benign” PI. The patients’ medical history was also remarkable for hypertension and COPD

Yet, the role and prognostic relevance of gas ascending into the liver, causing porto-mesenteric venous gas (PMVG) remains unclear. PMVG has been both described as linked to increased mortality as well as a reversible phenomenon [1, 10, 16–19] (Fig. 1). For most “unexperienced” radiologists and surgeons, the pattern of PI and gas in the portal venous system is indicative of a potentially irreversible intestinal infarction and associated with a poor prognosis. Although PI and PMVG have been discussed for decades, there is little to no comprehensive evidence-based data on PI and especially PMVG. Most data available on benign and reversible causes of PVMG is based on case reports or case series [10, 12].

Patients with PI and PMVG can have distinctly different prognoses. In clinical practice, it can be challenging to decide upon further treatment of patients with PI, even for experienced clinicians. The purpose of this study was to refine prognostication for these patients to improve decision-making in daily clinical routine with a special focus on patient outcome after surgical treatment.

## Methods and material

### Patients

**Fig.2:**
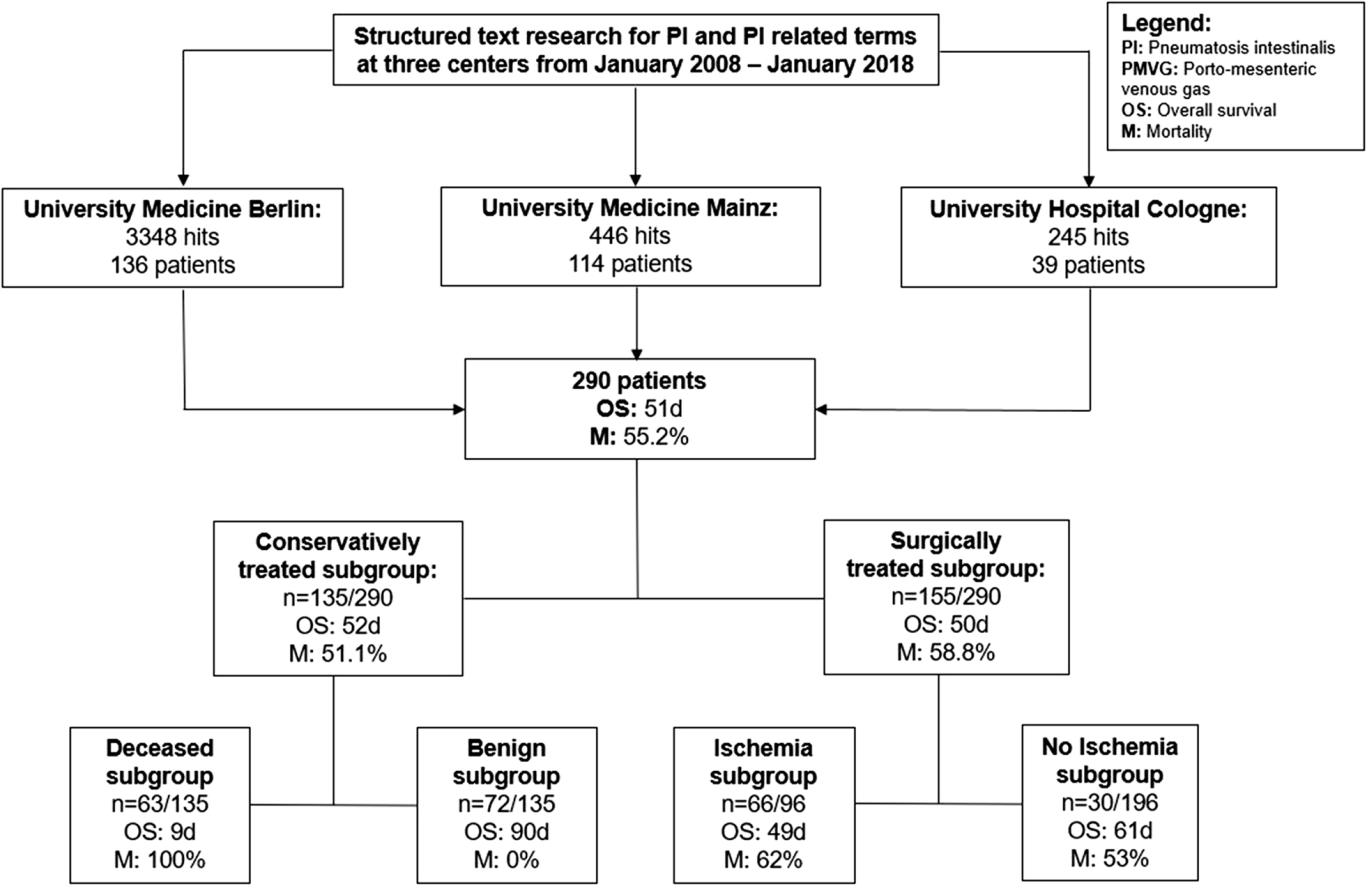
Flowchart showing the patient selection process, mortality and survival rates

Our institutional review board approved this retrospective multicentre study (registration number EA2/238/17). The study protocol conforms to the ethical guidelines of the 2002 Declaration of Helsinki. Informed consent was waived due to the retrospective nature of this study. The study was conducted at three tertiary care university medical centres (University Medicine Berlin, University Medical Centre Mainz, and University Hospital of Cologne). The respective radiological information systems (RIS) were searched from the readers for reports filed during the timespan of January 2008 to January 2018, and contained the following keywords: *pneumatosis, pneumatosis intestinalis, gas in the bowel wall, venous gas, gas in the portal vein and intramural gas *(Fig. 2). Where available, pathology and surgery reports were retrieved from the hospital information systems. The diagnosis of PI was confirmed by a board-certified radiologist after re-reading the respective imaging studies. To increase quality and to avoid false positives, all cases were re-read by two radiologists in consensus at every centre; one of each with at least ten years of abdominal CT experience (University Medicine Berlin: D.G and M.D.S; University Medical Centre Mainz: R.K and F.H; University Hospital of Cologne: D.P.D.S and J.H). Re-reading was performed to confirm the presence of gas within the gastrointestinal wall, as opposed to luminal gas, i.e. pseudo-pneumatosis phenomenon (false positive).

Mortality and overall survival (OS) were evaluated. OS was determined within a 90-day follow-up after the initial CT examination. To control for selection-bias, the following qualitative and quantitative risk factors were evaluated:

### Qualitative parameters

Age, sex (male and female), presence of PMVG, sepsis, vascular disease defined by varying degrees of stenosis (50–75%, 76–100%) of the superior or inferior mesenteric artery (AMS and AMI) or the coeliac trunk, underlying oncologic diagnosis, COPD, cardiovascular disease, presence of bowel obstruction (large vs. small bowel obstruction), decreased bowel wall enhancement and the therapeutic use of steroids.

### Quantitative parameters

Length of the PI-affected gastrointestinal segment (from 0 to 100 cm in increments of 10 cm), laboratory parameters including CRP, procalcitonin (PCT), platelet count, and lactate.

### Imaging acquisition

All patients were examined with multislice CT scanners and underwent single or multiphase CT examinations (arterial, and/or portal-venous and/or delayed phase). At the first institution (University Medicine Berlin) patients were either examined using a 128-multislice (Revolution HD and Revolution EVO, GE Healthcare, Milwaukee, WI, USA) or one of two 64-multislice CT scanners (Somatom Sensation 64, Siemens Medical Systems, Forchheim, Germany). All patients received a non-ionic intravenous contrast agent (either Ultravist 370, Bayer Schering, Berlin, Germany, Xenetix 350, Guerbet, Villepinte, France or Imeron 400, Bracco, Milan, Italy). At the second institution, patients were examined with either a 256-multisclice CT scanner or by a 64-multislice scanner (Philips iCT and Brilliance, Phillips, Amsterdam, Netherlands). All patients received a non-ionic intravenous contrast agent (Imeron 400, Bracco, Milan, Italy). At the third institution, patients were examined with either a 256-multislice CT scanner (Philips iCT, Philips, Amsterdam, Netherlands) or a 128-multislice scanner (Phillips IQon, Philips, Amsterdam, Netherlands). All patients received a non-ionic intravenous contrast agent (Accupaque 350, GE Healthcare, Milwaukee, WI, USA). Scan direction was craniocaudal in all institutions.

### Statistical analysis

All statistical analyses were performed using XLSTAT (Version 2011.0.01; Addinsoft SARL, New York, USA) and  with R 3.5.1 using RStudio 1.1.463; R Core Team (2018) [20]. Survival plots/Kaplan–Meier Curves were calculated and designed using R 3.5.1 with RStudio and help of the ‘survival’-package [21]. Contingency tables were used for statistical results with proportional distributions. OS was estimated using the Kaplan–Meier analysis and compared using the Logrank test. The Cox proportional hazard model was used for multivariate analyses to evaluate the influence of each risk factor on overall survival. A multivariate logistic regression model was used to test for influence of each risk factor on dichotomized variables (overall mortality, “benign” PI etc.). A p-value of less than 0.05 was considered statistically significant. Finally, a generalized linear / logistic regression model was built using the subset of patients who did not undergo surgery. We devised a statistical calculation model to predict death in surgically treated patients. A random sample of 100 patients from the conservatively treated cohort was used to fit the model. For random sampling a classified random sampling was performed using R’s “caret” package. The remaining 31 conservatively treated patients were used to verify the model’s performance. We then applied the model to the surgically treated patient cohort. We set an operating point for prediction of death with greater than 95% specificity to identify patients with an increased mortality risk despite surgical treatment. Accuracies were given as sensitivity, specificity, positive and negative predictive values (PPV/NPV).

## Results

### Demographics

A total of 290 patients (119 female and 171 male, mean age: 66.7, range: 18–92 years) with radiologically confirmed PI were finally included; concomitant PMVG was detected in 42% (122/290). PI was associated with radiologically suspected ischemia (decreased bowel wall enhancement) in 32.4% (94/290) and with mechanical (n = 43) or non-mechanical (n = 67) bowel dilatation in 38.0% (110/290). Of the patients with a mechanical bowel obstruction, 42% (18/43) had small bowel and 58% (25/43) large bowel obstruction. Mechanical bowel obstruction was observed significantly more often in the “PI only”- subgroup (***p* = *0.004***), while the location (small vs. large bowel) did not differ significantly. Sepsis was documented in 26.9% (78/290) of patients. Surgery was performed in 53.5% (155/290) of all patients (Fig. 1). Sepsis was significantly more prevalent in patients with coinciding PMVG (***p* = *0.010***), compared to the “PI only”-group, all other distributions showed no significant differences (Table 1).

**Table 1:** Descriptive patient characteristics (clinical parameters, overall mortality and pathologies)

Characteristics	Entire population (n = 290)	Subgroup—PI only (n = 168)	Subgroup—PI and PMVG (n = 122)	p-value
*Age—years*				
Mean—range	66.7—18–92	66.1—18–92	67.5—19–90	> *0.05*
*Gender (%)*				
Female	40.3 *(119)*	44.6 (75)	36.1 (44)	
Male	59.7 *(171)*	55.4 (93)	63.9 (78)	> *0.149*
Overall mortality (%)	55.2 *(160)*	46.5 (78)	67.2 (82)	
90d follow up (d)	51	58	41	** < *0.001***
*Subgroup analyses*				
Surgery cohort (%)	58.5 *(97)*	54.1 (46)	64.4 (45)	
90d follow up	50	56	43	*0.096*
Conservatively treated cohort (%)	51.1 *(63)*	38.6 (32)	71.1 (37)	
90d follow up (d)	52	61	37	** < *0.001***
*Aetiology (%)*				
Ischemia	32.4 *(94)*	30.1 (52)	34.4 (42)	*0.611*
Bowel obstruction	38.0 *(110)*	53.6 (59)	46.4 (51)	*0.393*
Mechanic	40.0 *(43)*	52.5 (31)	23.5 (12)	***0.004***
Small bowel	42.0 (18)	32.3 (10)	66.6 (8)	*0.058*
Large bowl	58.0 (25)	67.7 (21)	33.3 (4)	*0.058*
Non-mechanic	60.5 *(67)*	47.5 (28)	66.5 (39)	***0.004***
Sepsis	26.9 *(78)*	21.4 (36)	34.4 (42)	***0.016***

*p* < 0.05 was considered statistically significant

PI: pneumatosis intestinalis; PMVG: porto-mesenteric venous gas; OP: Operation

### All patients

**Fig. 3:**
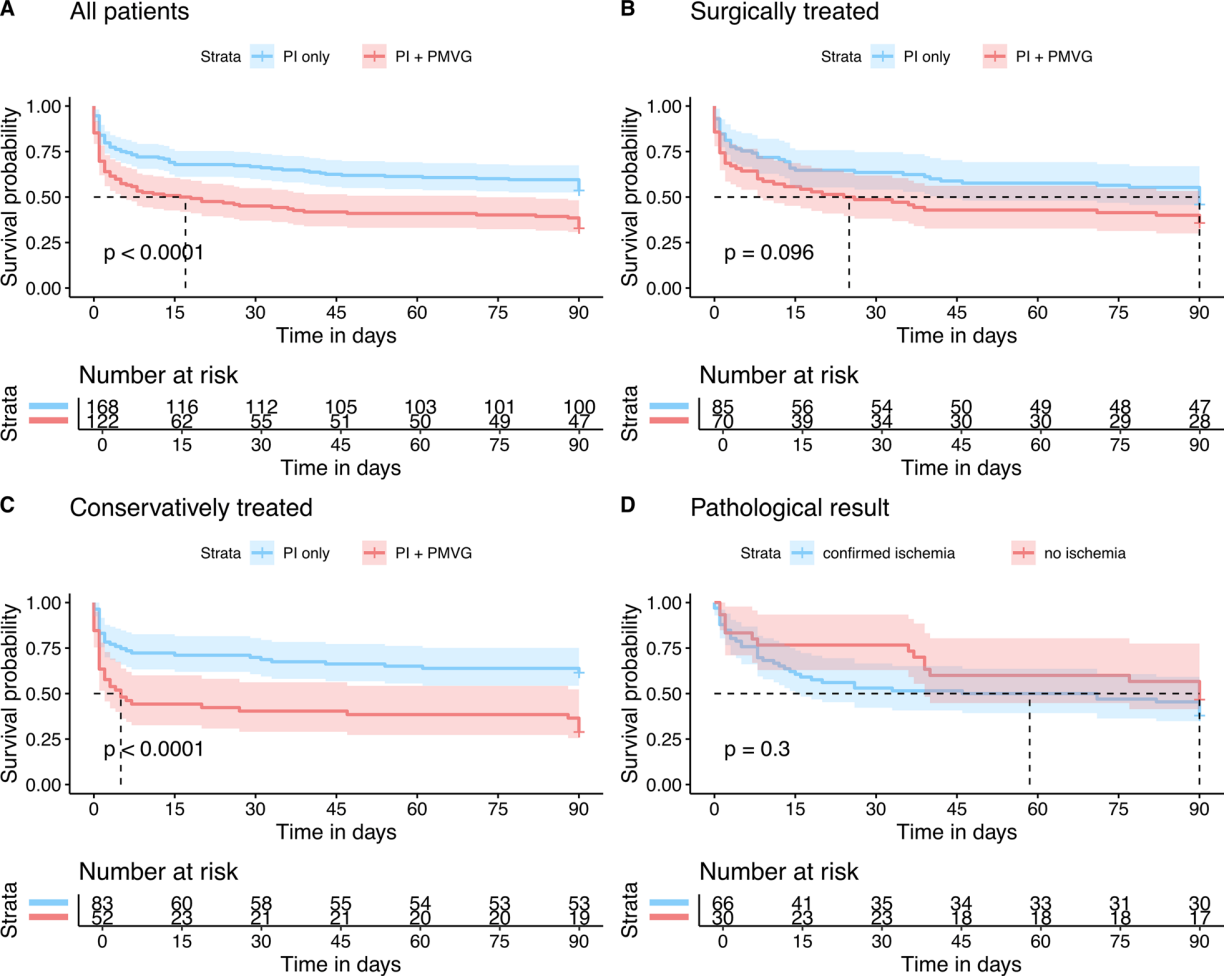
Primary endpoint 90d follow-up in days for **A** the entire collective and patients with “PI only” (blue) and “PI + PMVG” (red); **B** surgically treated patients with “PI only” (blue) and “PI + PMVG” (red); **C** conservatively treated patients with “PI only” (blue) and “PI + PMVG” (red); and **D** the pathologically confirmed subgroup “with ischemia” (blue) and patients “without ischemia” (red)

Overall mortality for all patients within the 90d follow-up was 55.2% (160/290; median OS 51d; 2.3 ± SD; 95% CI 46.1–55.7). Mortality was 46.5% (78/168) in patients with PI only and increased significantly to 67.2% (82/122) in the presence of PMVG (***p* < *0.001***) (Fig. 2). Accordingly, Kaplan–Meier analyses showed a significantly longer OS for patients with PI only (58d ± 3.1; 95% CI 52.4–64.5) compared to patients with PI and PMVG (41d ± 3.8; 95% CI 33.1–48.0) (*p* < *0.001*) (Fig. 3).

### Subgroup analyses

#### Surgical treatment

The collective was further subdivided into subgroups depending on treatment. Surgery was performed in 53.5% of all patients (155/290), mortality in this cohort was 58.8% (91/155) (50d ± 3.3; 95% CI 44.0–56.5). In patients with PI only (85/155) mortality was 54.1% (46/85) (median OS 56d ± 4.4 SD; 95% CI 47.1–64.4) and increased to 64.3% (45/70) (median OS 43d ± 5.0 SD; 95% CI 33.3–56.7) in the presence of PMVG (155/290) (***p* < 0.096**). A pathology report was available in 62.0% (96/155) of these surgically treated patients. Intestinal ischemia was diagnosed in 67.5% (66/96), while in 32.5% (30/96) the pathologist found no sign of ischemia or intestinal infarction. Mortality and OS did not differ significantly between these subgroups (ischemia subgroup: 49d ± 5.0 SD; 95% CI 39.8–59.0) vs. non-ischemia subgroup: 61d ± 6.9 SD; 95% CI 37.1–74.3) (*p* = *0.304*) (Fig. 3).

#### Conservative treatment

46.5% of all patients (135/290) were managed conservatively (i.e. without surgery). Mortality in this cohort was 51.1% (69/135) (52d ± 3.7; 95% CI 44.8–59.2). In patients with PI only mortality was 38.6% (32/83) (median OS 61d ± 4.4 SD; 95% CI 52.6–69.9) and increased significantly to 71.2% (37/52) (median OS 37d ± 5.9 SD; 95% CI 25.750.0) in the presence of PMVG (39/55) (***p* < 0.001**). Patients were further divided in two different subgroups. The first included patients who were treated conservatively and survived the 90d follow-up (n = 72) (“benign” cohort). The second group included patients who were unfit for surgery and died within the 90d follow-up (n = 63) (“deceased” cohort). Median survival for patients who died within the 90d follow-up was 9d ± 2.1 (SD; 95% CI 4.7–13.1). The prevalence of PMVG was significantly higher (**p < 0.05**) in the “deceased” cohort 52.2% (33/63) compared to the “benign” cohort (27%; 19/72) (Figs. 2 and 3). Detailed results can be found in Table 1.

#### Comprehensive risk profile analysis

In a second step, the possible influence of several risk factors on survival were calculated by means of multivariate regression models (Table 2).

**Table 2:** Comprehensive multivariate risk factor analysis

Risk factor / parameters		Cox-Regression		MultivariateRegression		MultivariateRegression
	Entire population(n = 290)	*p-value*	Benign subgroup(n = 72)	*p-value*	Deceased subgroup(n = 63)	*p-value*
*Qualitative parameters:*						
Sex		*0.626*		*0.218*		*0.464*
Female	119		26		23	
Male	171		46		40	
PMVG (*present):*	122	*0.161*	19	***0.042***	33	*0.486*
Sepsis (*present):*	78	***0.005***	10	***0.033***		*0.108*
Vascular disorders: (present)	100		17			
50–75%	28	*0.775*	8	*0.559*	5	*0.384*
76–99%	34	***0.010***	4	*0.467*	8	*0.447*
Occlusion	28	*0.137*	5	*0.369*	10	*0.959*
Oncologic disease *(present)*	110	*0.767*	34	*0.268*	23	*0.464*
Cardiovasc. disease *(present)*	200	*0.685*	40	*0.253*	42	*0.772*
COPD (*present)*		***0.002***	7	***0.017***		*0.715*
Bowel-obstruction: (*present*)	109	*0.766*	23	*0.262*	26	*0.689*
Steroids therapy*(present)*	28	*0.314*	7	*0.182*	3	*0.182*
Length						
10-90 cm	236	*0.097–0.904*	62	***0.039***	45	***0.039***
> 100 cm	54	***0.007***	10	***0.039***	18	***0.039***
*Quantitative parameters*						
Age	66.7 ± *14.8*	***0.041***	66.0 ± *14.7*	*0.647*	64.6 ± *16.1*	*0.654*
CRP (mg/l)	129.4 ± *125.6*	***0.008***	80.3 ± *96.5*	***0.006***	136.9 ± *118.9*	*0.555*
PCT (μg/l)	8.2 ± *6.1*	*0.053*	2.7 ± *4.7*	*0.146*	11.7 ± *15.0*	*0.339*
Lactate	32.6 ± *39.9*	***0.042***	26.5 ± *38.0*	*0.689*	46 ± *63.9*	*0.540*
Platelets	218.1 ± *141.6*	** < *0.001***	231.7 ± 116.2	*0.635*	122.8 ± *115.0*	** < *0.001***

*p* < 0.05 was considered statistically significant

#### Cox proportional hazard model

The following parameters were associated with decreased overall survival: old age (***p* = *0.041***; HR: 1.01 / 95% CI 1.00–1.03); sepsis (***p* = *0.005***; HR: 1.82 / 95% CI 1.20–2.75); coeliac/mesenteric artery stenosis of 76–99% (***p* = *0.010***; HR: 2.00 / 95% CI 1.17–3.26), COPD (***p* = *0.002***; HR: 1.92/95% CI 1.28–2.87), length of intestine affected by PI > 1 m (***p* = *0.007***; HR: 2.07 / 95% CI 1.25–3.50), elevated CRP-count (***p* = *0.008***; HR: 1.00 / 95% CI 1.00–1.00), elevated serum lactate levels (***p* = *0.042***; HR: 1.00 / 95% CI 1.00–1.05), and decreased platelet count (***p* < *0.001***; HR: 1.00 / 95% CI 1.00–1.01). Furthermore, elevated PCT showed a trend towards shortened survival but did not reach statistical significance (*p* = *0.053*, HR: 1.01 / 95% CI 1.00–1.03) (Table 2).

#### Multivariate logistic regression model

The following parameters were associated with reversible / “benign” PI: the absence of PMVG (***p* < *0.042***), the absence of COPD *(****p* < *0.017***) and low CRP levels (***p* = *0.006***). All other risk factors had no significant influence on reversibility of PI in the conservatively managed group. A low platelet count was found to be associated with fatal outcome with the group of patients who were managed conservatively/i.e. non-surgically (***p* < *0.001).***

#### Multivariate estimation model

As abovementioned, the following parameters were statistically significant for a decreased OS: PMVG, COPD, sepsis and low platelet count.

All other results and parameters out of the multivariate risk factor analysis are displayed in Table 2*.*

**Fig. 4:**
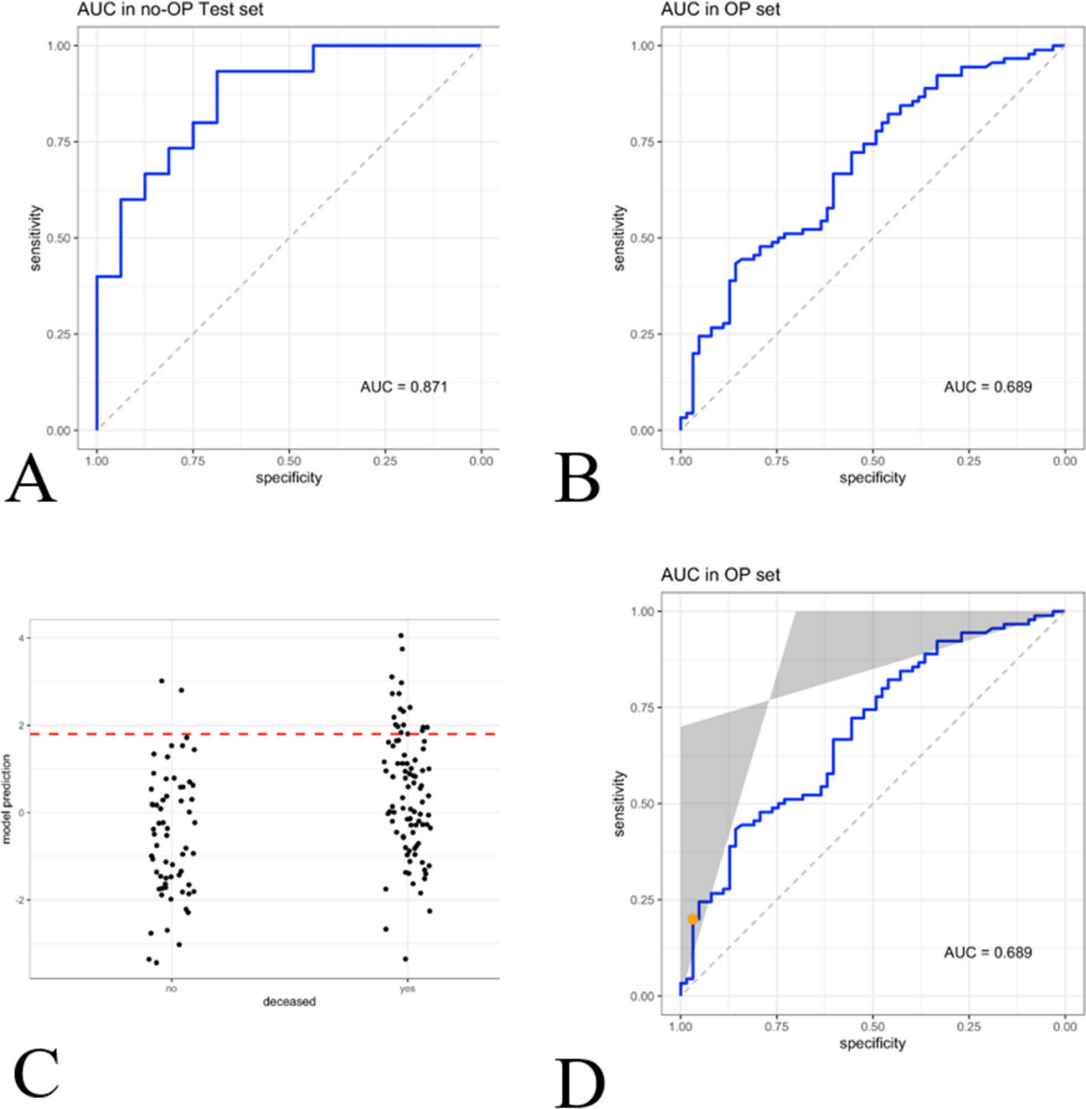
Calculation of the generalized linear / regression model using a subgroup of patients who were managed conservatively (Test set—**A**). Further evaluation by applying the model to the subgroup treated surgically (OP set—**B**). To design a decision support tool, we estimated an operating point for prediction of death with greater than 95% specificity (**C**, **D**)

After subdividing patients into two cohorts, a surgically treated and conservatively treated cohort, in the no-surgery cohort, the parameters were re-tested again by means of a training set to predict death. P-values generated from the training set were as follows: PMVG: ***p* = *0.023***, COPD: ***p* = *0.083***, sepsis: ***p* = *0.006*** and thrombocytes: ***p* = *0.003–****AUC: 0.818* (Fig. 4A). In a second step, we used the model to analyse the surgery-cohort with these parameters to predict death and calculated an AUC of 0.689 (Fig. 4B). We identified and defined a point on the curve with a specificity of 95%. To reach this specificity a sensitivity of 20% had to be accepted with a threshold of 1.8 (Fig. 4C). Accuracies generated by this model to predict death were as follows: 97% specificity, 20% sensitivity, PPV 90%, NPV 45%. When dividing the cohorts according to the calculated threshold, resulting Kaplan–Meier curves also showed significant differences in overall survival (***p* < *0.001***) (Fig. 4D).

## Discussion

Prognostication in patients with PI and PMVG is challenging. This study confirms PI as a life-threatening diagnosis with an associated mortality rate of 54.1%. In the presence of PI and concomitant PVMG, mortality increases to 68.2%. Especially the presence of infection and ischemia correlate with a worse prognosis. Nevertheless, patients with “benign”, i.e. reversible PI are common (25% in this study). The mathematical model we introduce helped us to pinpoint risk factors (PMVG, COPD, platelet count, sepsis) to identify patients with an increased mortality risk.

**Fig. 5:**
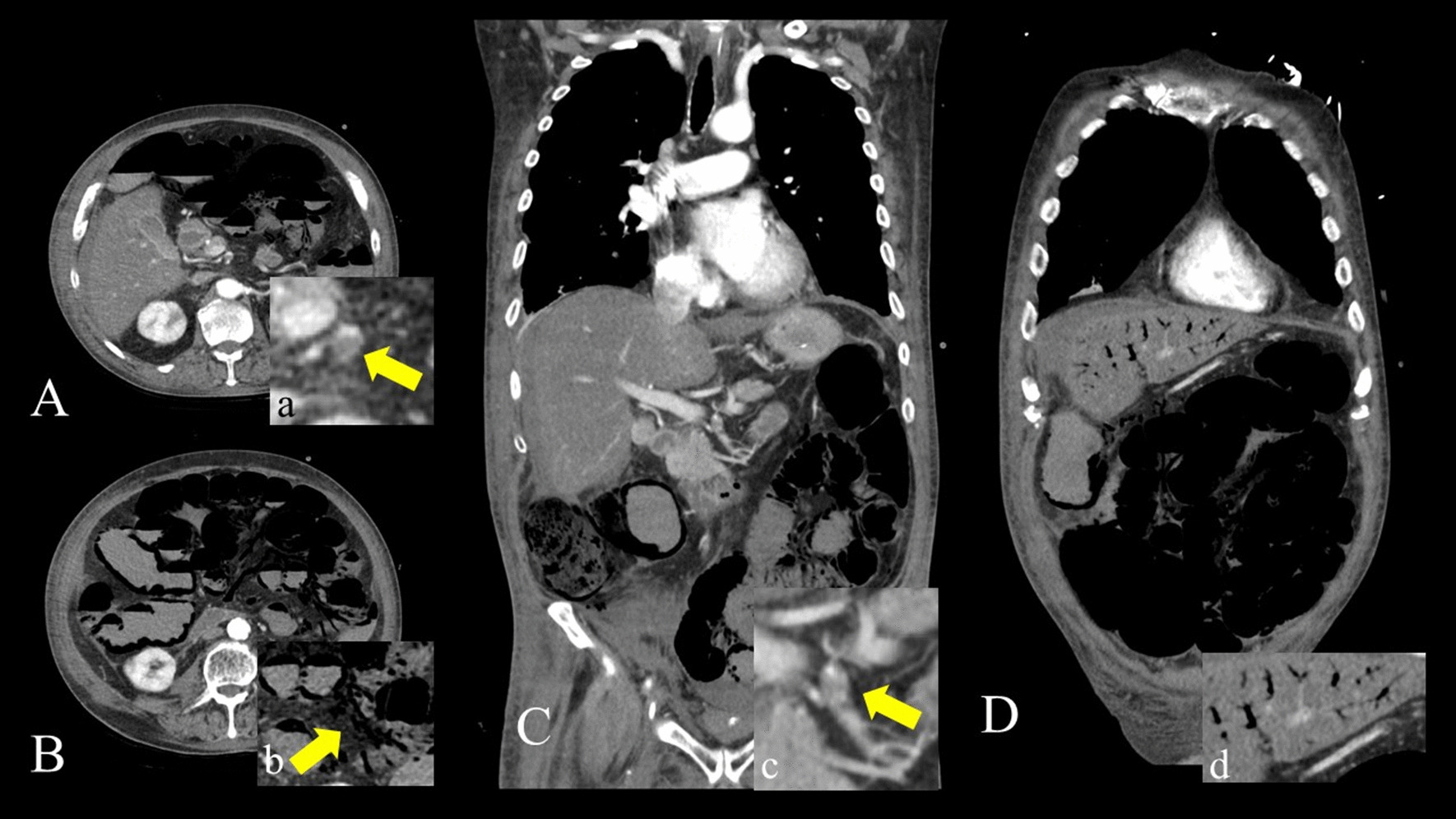
A-D is showing an arterial phase contrast enhanced CT with axial slices (**A**, **B**) and coronar reconstructions with an extensive PI and PMVG in a 68-year-old patient with an acute embolism (yellow arrow in **c**) of the superior mesenteric artery (**A**, **C**) suffering from ischemia of the small bowel, transverse and ascending colon. Apart from cystic air bubbles within the intestinal wall, note the presence of gas in the mesenteric veins (PVMG) (**B**/**b**) and portal veins of the left upper liver lobe (**D**/**d**). Explorative laparotomy showed extensive intestinal necrosis. The patient died within 24 h after the CT-examination

**Fig. 6:**
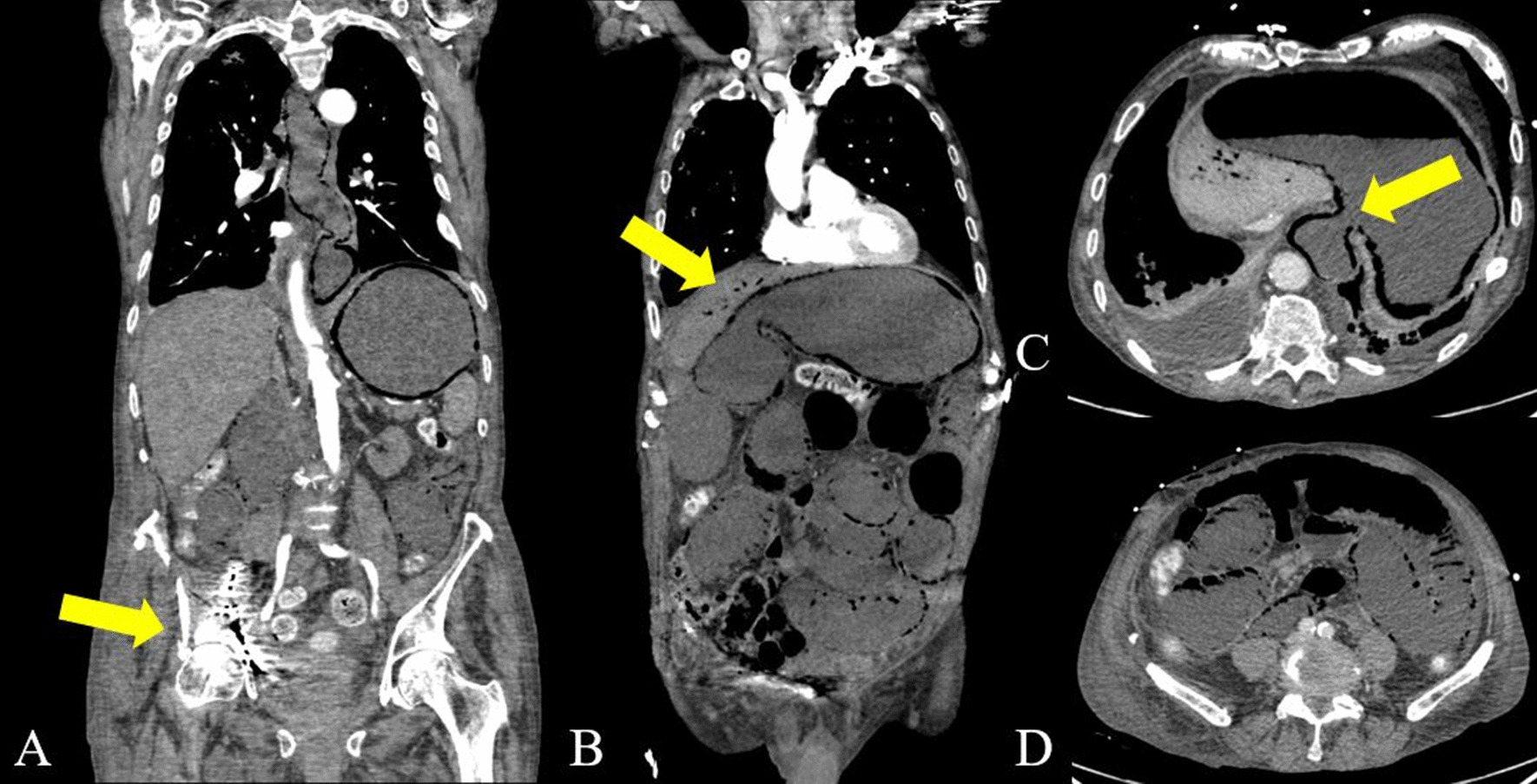
A-D is showing an arterial phase contrast enhanced CT with axial slices (**A**, **B**) and coronar reconstructions with a fulminant PI and PMVG (**B**, **D**) in a 85 year-old patient with sepsis. Note the presence of gas within in the intestinal wall the entire small and large intestine, as well as the oesophagus (**C**), caused by bacterial enteritis after surgical fixation of an acetabular hip fracture on the right (**A**) with pelvic perforation of the osteosynthesis. The patient died within 24 h after the CT-examination

While the true incidence of PI is unknown, PI is diagnosed in day-to-day radiologic routine and its prognostic value remains unclear; especially in a setting with coincident PVMG. Some case reports have been published claiming that PMVG might have reversible causes, while others suggest PMVG has a dismal prognosis [22]. The physical examination of the patients plays a key role in management and triage, as patients with signs of peritonitis or suspicion of intestinal ischemia and perforation are candidates for explorative surgery. However, other parameters to assist clinical decision-making and prognostic factors to potentially differentiate fatal from benign/ reversible PI are currently lacking. There are some factors, such as elevated serum lactate that seem to be associated with an increased mortality, mainly due to the worse outcome in patients with ischemia, albeit with a low level of evidence (Figs. 5A–D and 6A–D) [23–25].

With 290 patients, this multicentre trial is one of the largest of its kind since the introduction of CT scanners. The mortality rates described in this study are in line with current studies. Lassandro et al. reported a 30.4% overall mortality for PI and 50% for PI in combination with PMVG in 102 patients [26]. Treyaud et al. reported an overall mortality of 41.6% in 149 patients. We found that the presence of PMVG correlated with ischemia, which is in line with Treyaud et al. However, Treyaud et al. defined decreased bowel wall enhancement on CT as “ischemia”, if—like in most cases—pathology reports were not available [22]. In our study, the pathology report was used as the gold standard for ischemia, which we then used to correlate ischemia with the presence of PMVG.

Despite the high mortality rates reported in this study, and the fact that survival was even lower in the presence of PMVG, we identified 72 patients who survived with non-surgical management. Although the prevalence of PMVG in this subgroup was low (10%), it was diagnosed in a few cases. This suggests that, PI in coincidence with PMVG can also have benign and reversible causes, and both radiologists and surgeons should be familiar with these [15], highlighting the need for further large-scale studies to identify the aetiology of “benign” / i.e. reversible PI and PMVG. Subsequently, we evaluated a comprehensive risk profile for each patient retrospectively to understand and investigate factors associated with increased mortality as opposed to “benign”/reversible cases. First, we analysed factors associated with decreased survival rates. In line with the literature, we found elevated serum lactate levels to be associated with a worse prognosis [23–25]. Old age, sepsis and an arterial stenosis were also found to correlate with shortened OS. Hence, we conclude that intestinal infarction, which is also linked to ischemia, remains the most life-threatening diagnosis in case of radiologically confirmed PI. Surprisingly, the strongest statistical correlation between shortened survival and the only significant factor to occur in patients who deceased without surgery, was a low platelet count. Platelets play a central role in the regulation of blood flow and thrombogenic cascades [27]. An increased platelet count was thought to be directly associated with increased vascular inflammation and thrombogenicity and therefore to correlate with thrombotic events like ischemic stroke and myocardial infarction [27, 28]. Conversely, in this study, a low platelet count was associated with a worse prognosis. It is possibly even more interesting to investigate factors that correlate with “benign” PI to identify those patients who do not require surgery but conservative treatment. In our study, low CRP levels, the absence of PMVG, sepsis and COPD were predictive for reversible PI.

When combining the significant parameters identified in the logistic regression model (PMVG, COPD, sepsis and low platelets) to predict a fatal outcome in surgically treated patients, our multivariate model reached a specificity of over 90%. This statistically validated risk profile could serve as a decision support tool to identify patients who are unlikely to benefit from surgery, and thus potentially reducing overtreatment.

### Limitations

Our study has several limitations. First and foremost, the retrospective study design and the fact that all readers were aware of this design, introduce a potential detection bias. Secondly, there is an inherent selection bias, as the RIS were searched for reports containing PI and PI-related terms. Hence, if none of these terms were mentioned in the radiology report, the patient was not included. As a diagnosis of PI does not require an arterial contrast phase, not all of the included patients received a CT scan comprising that phase. This can limit the chances of detecting decreased bowel wall enhancement and therefore explain why ischemia did not significantly correlate to the pathology results in all patients undergoing surgery. Also, the medical history was incomplete in some patients. Although pathological reports were available in surgically treated patients they did not provide 100% specificity, which is a further potential cause of distortion.

## Conclusion

PI is not necessarily associated with intestinal ischemia but may be attributed to various other causes. The coincidence of PI and PMVG remains indicative of intestinal ischemia and strongly correlates with significantly increased mortality rates. The validated risk profile we introduce can serve as an ancillary decision-making tool to identify patients who are unlikely to benefit from surgery and to potentially reduce overtreatment.

## Data Availability

The datasets used and/or analysed during the current study available from the corresponding author on reasonable request.

## Abbreviations

CRP: C-reactive protein
COPD: Chronic obstructive lung disease
CT: Computed tomography
IMA: Inferior mesenteric artery
OS: Overall Survival
PCT: Procalcitonin
PI: Pneumatosis intestinalis
PMVG: Porto-mesenteric venous gas
RIS: Radiological information system
SMA: Superior mesenteric artery
